# Serological evidence of tick-borne Crimean-Congo haemorrhagic fever and Dugbe orthonairovirus infections in cattle in Kwara State in northern Nigeria indicate independent endemics

**DOI:** 10.1371/journal.pntd.0012539

**Published:** 2024-10-21

**Authors:** Oluwafemi Babatunde Daodu, Julia Hartlaub, James Olukayode Olopade, Daniel Oladimeji Oluwayelu, Martin H. Groschup

**Affiliations:** 1 Department of Veterinary Microbiology, Faculty of Veterinary Medicine, University of Ilorin, Ilorin, Nigeria; 2 Institute of Novel and Emerging Infectious Diseases, Friedrich-Loeffler-Institut, Greifswald, Insel Riems, Germany; 3 Department of Veterinary Anatomy, Faculty of Veterinary Medicine, University of Ibadan, Ibadan, Nigeria; 4 Department of Veterinary Microbiology, Faculty of Veterinary Medicine, University of Ibadan, Ibadan, Nigeria; 5 Center for Control and Prevention of Zoonoses, Faculty of Veterinary Medicine, University of Ibadan, Ibadan, Nigeria; Public Health Agency of Canada, CANADA

## Abstract

Crimean-Congo haemorrhagic fever orthonairovirus (CCHFV) and Dugbe orthonairovirus (DUGV) are zoonotic viruses transmitted by ticks. Whereas CCHFV has caused numerous human cases, DUGV, although less reported, shares ticks and ruminants as hosts. Since its first discovery in Nigeria in 1964, there has been no detailed sero-epidemiological investigation on DUGV in sub-Saharan Africa. This study is aimed at assessing the current seroprevalence and associated risk factors of CCHFV and DUGV infections in Nigerian cattle. Using a cross-sectional design with random sampling method, blood samples were collected from 877 cattle on pastoralist farms and at abattoirs in Kwara State, North-Central Nigeria. CCHFV IgG antibodies were detected in extracted sera using three panels of in-house indirect enzyme-linked immunosorbent assay (ELISA) based on bacteria-expressed recombinant nucleoprotein (rNP), the cattle-adapted VectoCrimean ELISA and the ID Screen CCHF double antigen multi-species ELISA, while DUGV IgG antibodies were detected using in-house indirect ELISA with bacteria-expressed rNP, indirect immunofluorescence assay and micro-Virus Neutralization test. Overall seroprevalence rates of 71.9% (631/877) and 52.8% (451/854) were obtained for CCHFV and DUGV, respectively. It was observed that 37.9% (314/829) of the cattle were co-exposed to both CCHFV and DUGV while 34.5% (286/829), 14.8% (123/829) and 12.8% (106/829) were exposed to single infections with CCHFV, DUGV or none of the two viruses, respectively. Multivariate analysis showed that only location, sex, age and tick infestation score were the risk factors that significantly affected CCHFV seroprevalence in cattle, while DUGV seroprevalence was significantly influenced by month of the year, location, cattle breed and sex (p<0.05). This is the first comprehensive sero-epidemiological surveillance for DUGV in sub-Saharan Africa. Our findings reveal widely distributed independent CCHFV and DUGV infections in cattle in Kwara State, Nigeria.

## Author summary

Crimean-Congo hemorrhagic fever orthonairovirus (CCHFV) and Dugbe orthonairovirus (DUGV) are viruses that can be transmitted to humans through ticks. While CCHFV has been known to cause several cases of illness in humans, DUGV, which is distantly related to CCHFV, has been associated with fewer cases. Both viruses are commonly found in ticks and animals like cattle. This study was aimed at determining the level of exposure of cattle in Kwara State, Nigeria to these viruses through antibody detection. Blood samples collected from 877 cattle on farms and in abattoirs in Kwara State, Nigeria, were tested for antibodies to both CCHFV and DUGV. The results revealed that a significant proportion of the sampled cattle had antibodies to both viruses, indicating previous exposure. Specifically, 71.9% (631/877) of the cattle tested positive for CCHFV antibodies, while 52.8% (451/854) tested positive for DUGV antibodies. Furthermore, 37.9% (314/829) of the cattle were co-exposed to both CCHFV and DUGV, while 34.5% (286/829) had been exposed only to CCHFV, 14.8% (123/829) only to DUGV, and 12.8% (106/829) to neither virus. Multivariate analysis revealed that only location, sex, age, and tick infestation score significantly affected CCHFV seroprevalence, while for DUGV, month of the year, location, cattle breed, and sex were observed to significantly influence seroprevalence (p<0.05). This study provides the first comprehensive report on DUGV presence in sub-Saharan Africa, indicating that both CCHFV and DUGV are present in Kwara State, Nigeria.

## Introduction

Crimean-Congo haemorrhagic fever orthonairovirus (CCHFV) is a highly contagious tick-borne zoonotic virus causing fever, malaise, haemorrhagic disorder, and may lead to death. A distantly related virus, Dugbe orthonairovirus (DUGV), was first isolated in Nigeria [1] and has once been reported to cause disease in humans with pyrexia, haemorrhage and decreased platelet count [2]. These viruses have been classified as Biosafety Level 3 (DUGV) and 4 (CCHFV) agents [3,4] because of their ease of spread and the severe diseases they cause in humans. Infections with these viruses in animals are subclinical and infected ticks cannot be identified with deranged physiology, thus making it difficult for humans working with animals to avoid infected animals and ticks. However, arboviral exposure in animals has been suggested as an indicator of arboviral spread in the environment and also qualifies as a tool for defining humans at risk [2].

Crimean-Congo haemorrhagic fever and Dugbe orthonairoviruses are members of the genus *Orthonairovirus*, family *Nairoviridae* and order *Bunyavirales*. They are enveloped viruses with tripartite negative sense, single-stranded RNA genome. Although CCHFV and DUGV belong to the same genogroup (Nairobi Sheep Disease group) [5,6], serologically, CCHFV belongs to the Crimean-Congo haemorrhagic fever serogroup while DUGV belongs to the Nairobi sheep disease serogroup [7].

The occurrence of tick-borne bacterial and protozoal infections including those caused by *Rickettsia* spp., *Borrelia* spp., *Theileria* spp., *Babesia* spp., *Anaplasma* spp., *Ehrlichia* spp. and *Coxiella burnetii* have been documented in Nigeria [8,9]. However, information on tick-borne viral diseases is limited, with their current prevalence and distribution remaining largely unknown following the earlier studies conducted four to five decades ago. Specifically, although animal exposure to CCHFV is well reported in countries of Europe and Asia, there is still scanty data on the disease and assessment of epidemiological risk factors that influence its occurrence among animals in Nigeria. Also, information on the current impact of DUGV, a neglected arbovirus, on animal and human health in Nigeria remains largely unavailable. This study was therefore designed to assess the seroprevalence of CCHFV and DUGV infections and their associated risk factors among cattle in Kwara State, North-central Nigeria.

## Materials and methods

### Ethics statement

Ethical approvals for this study were issued by the Animal Care and Use Research Ethics Committee (ACUREC) of the University of Ibadan (UI-ACUREC/092-1121/18) and Kwara State Ministry of Agriculture and Natural resources (VKW-714/I/83). Also, approval and verbal consent of the animal owners were obtained before the commencement of sampling.

### Study area, sampling sites and design

The study location was Kwara State of Nigeria. It is the busiest transit State for ruminant transportation from the Northern to the Southern region of Nigeria. The State has both forest and savannah vegetation. The abundant grass land and water bodies in the State provide adequate grazing land to resident ruminants as well as the migratory ones from the north during the dry season. Ten out of the 16 local government areas (LGAs) in the State were randomly selected for this study. Subsequently, 31 sampling sites were arbitrarily selected with 27 pastoralist farms and four major abattoirs. A cross-sectional design was adopted while cattle in farms and abattoirs were randomly selected for sample collection. The sampling period spanned between February 2017 to March 2018.

### Sample collection and processing

Blood (5 ml) was obtained aseptically from each cattle through jugular venepuncture and dispensed into plain tubes. These were then transported to the laboratory (under cold chain conditions) and centrifuged at 1500 revolutions per minute for 10 minutes. Sera were separated into cryovial tubes and stored at -20°C until used.

### Data collection

The demographic information of the cattle sampled were collected by physical examination of the animal and history taken from their owners. These include sex, age, management system, and body condition score (BCS). The BCS of individual cattle is classified into four categories as Poor (animals that are weak, recumbent, emaciated with prominent transverse processes, and dehydrated), fair (animals that are weak, recumbent and emaciated with prominent transverse processes), good (animal is active without prominent bones, has shiny hair coat and is well fleshed, dorsal spines can be felt with firm pressure but feel rounded rather than sharp), and very good (animal is active with heavy deposits of fat on tail-head, brisket and cod; dorsal spines, ribs, hooks and pins fully covered and cannot be felt even with firm pressure)[10].

### Serological assays

#### Detection of anti-CCHFV IgG antibodies

CCHFV IgG detection in cattle sera was done using three panels of ELISA tests (FLI CCHFV in-house indirect ELISA, cattle-adapted VectoCrimean ELISA (VectorBest, Novosibirsk, Russia) and ID Screen CCHF double antigen multi-species ELISA (IDVet, Grables, France)).

#### FLI CCHFV In-house Indirect ELISA

This ELISA was performed using microtitre plates coated with CCHFV Kosovo Hoti recombinant nucleoprotein (KH-N-Protein) [11]. Each well of rows A, B, E and F of Sterile Flat bottom microtiter (Greiner F plate) was coated with 100 μl of diluted laboratory prepared KH-N-Protein at 0.2 μg/well in coating buffer (0.5% of Albumin fraction V in Phosphate Buffer Saline at pH 9) while rows C, D, G and H were filled with 100 μl of coating buffer (without KH-N-Protein). The plate was incubated in the fridge (4°C) overnight for up to 60 hours. The content of the well was discarded, and the plate was pushed on cellulose to completely remove the liquid droplets. Subsequently, 200 μl/well of blocking buffer (IDvet, France) was added to each well and incubated at 37°C for 1 hour. The content of the well was discarded, and the plate was again blotted on cellulose. The test samples and controls sera (positive control serum from experimentally infected animal at Friedrich Loeffler Institut, Germany) were then diluted 1:40 using dilution buffer 11 (IDvet, France) and 100 μl was dispensed into four wells each. Thereafter, the plate was incubated for 1 hour at 37°C, after which it was washed (0.1% Tween 20 in phosphate buffered saline) three times using 250 μl/well and blotted on cellulose. Subsequently, 100 μl of 1:1000 diluted goat anti-bovine-IgG horseradish peroxidase conjugate (Southern Biotech, USA) was dispensed into each well and incubated for 2 hours at 37°C. The washing and blotting steps were then repeated. Later, 100 μl/well 3, 3´, 5, 5´-tetramethylbenzidine (TMB) (Bio-Rad, Germany) was added to each well and incubated in the dark at room temperature for 15 minutes. Finally, the reaction was stopped by addition of 100 μl 1M H_2_SO_4_ per well. The plate was read at 450 nm (620 nm reference emission).

For each sample, the corrected OD value was calculated as ((A+B)/2-(C+D)/2). The test is said to be valid if the corrected OD value of the positive control is over 1 and the negative control is negative. The final result was calculated as the percentage of the positive control (i.e., corrected OD value of the sample divided by corrected OD value of positive control multiplied by 100). The ELISA test percentage negative cut-off and positive cut-off were set at < 16% and > 19%, respectively.

#### VectoCrimean CCHFV ELISA

This test was performed with a microtiter plate coated with CCHFV KH-N-Protein, after the protocol described by Mertens et al [12]. Briefly, test and control sera were each diluted 1:100 using 1:10 SPSD/SDB (supplied with the kit) as diluent. Then, 100 μl of the diluted serum was dispensed into the coated plate and incubated for 1 hour at 37°C. The plate was washed 5 times with 250 μl wash solution/well and blotted on paper towels to remove the remaining liquid droplets in the wells. Subsequently, 100 μl of 1:6000 diluted goat anti-bovine-IgG horseradish peroxidase conjugate in CDB (conjugate diluent, supplied with the kit) was dispensed into each well and incubated for 30 minutes at 37°C. The washing and blotting steps were then repeated after which 100 μl/well of substrate (TMB) was added to each well and the plate incubated in the dark at room temperature for 15 minutes. The reaction was stopped by addition of 100 μl/well stop solution. The plate was read at 450 nm (620 nm reference emission) and the difference of the extinctions was calculated.

The cut-off value of optical density (OD_co_) was obtained using the formula: 0.2 + OD_av_c^-^ where OD_av_c^-^ is the average of optical density of wells with negative control sera. Results were considered positive if OD sample is ≥ OD_co_ and negative if OD sample is < 0.8 x OD_co_.

#### ID Screen CCHF Double Antigen Multi-species ELISA

This ELISA was carried out using microtitre plates coated with Nigerian CCHFV strain (IbAr10200) nucleoprotein and following the manufacturer’s instructions. Briefly, 50 μl of dilution buffer 14 (supplied with the kit) was dispensed into all the wells of a ‘U-bottom’ microtiter plate (for dilution purpose). Then, 30 μl each of negative control sera was added to wells A1 and B1, while 30 μl each of positive control sera were added into wells C1 and D1. The remaining wells were filled with 30 μl of each test serum. The diluted sera were then transferred into antigen-coated plates and left to incubate at room temperature (21°C ± 5°C) for 45 ± 4 minutes. The plate was emptied, washed 5 times with 300 μl wash solution/well and later blotted on paper towel to remove the remaining liquid in the wells. Subsequently, 50 μl 1X conjugate was added to each well of the plate and incubated at room temperature for 30 ± 3 minutes. The plate was emptied and washed as previously described. Thereafter, 100 μl of substrate solution (TMB) was added to each well and the plate was incubated in the dark for 15 ± 2 minutes at room temperature. Finally, 100 μl of stop solution was added to each of the wells. The plate was read at 450 nm and OD values recorded. The test was considered valid if the mean OD value of the positive control serum (OD_PC_) was greater than 0.35 and the ratio of the mean OD values of the positive (OD_PC_) and negative (OD_NC_) control sera were greater than 3 i.e., OD_PC_/ OD_NC_ > 3.

The percentage Sample-to-Positive control ratio (S/P%) was calculated as OD_sample_ divided by OD_PC_ and then multiplied by 100. Samples presenting S/P% > 30% were considered positive while those with S/P% ≤ 30% were considered negative.

#### Interpretation of CCHFV serology results

Each individual assays conducted could stand alone for prevalence determination. However, in order to increase the accuracy of the seroprevalence, results for each sample were interpreted based on agreement among the ELISA tests conducted (a sample is considered to be positive or negative if all the three assays agree; if one or more of the ELISA tests were not in agreement, the tests were repeated for that particular sample and if disagreement were to persist, the divergent result were presented as doubtful). We had previously ensured that our serological assays could discriminate between CCHFV and DUGV antibodies [13]. Thus, the selection of assays to be used was pre-informed such that it was only when these assays were in agreement that the result was interpreted as positive or negative, otherwise, it was recorded as doubtful.

### Detection of anti-DUGV IgG antibodies

Three serological assays were performed per sample for DUGV antibody detection (indirect IgG ELISA, indirect immunofluorescence assay and micro-virus neutralization test).

#### Indirect IgG Enzyme-linked Immunosorbent Assay

A bacterially expressed recombinant nucleoprotein of DUGV (DUGV N protein) was used as plate-coating antigen (2 μg/ml in coating buffer) [13]. Each sample was tested in two wells coated with antigen and without antigen (i.e., having only dilution buffer). The plate was incubated at 37°C for 1 hour, followed by a washing step which was repeated 3 times. Thereafter, 200 μl/well of blocking buffer (IDVet, Grabels, France) was added with incubation at room temperature for 1 hour. Subsequently, 100 μl serum (diluted 1:20 in IDVet Buffer No. 3) was added to each well, the plate incubated for 1 hour at 37°C, and then washed three times. The plate was eventually incubated (30 minutes at 37°C) with horseradish peroxidase-conjugated goat anti-bovine IgG (diluted 1:10,000 in IDVet Buffer No. 11) and washed three times. The substrate (TMB) was then added and incubated for 10 minutes at room temperature in the dark. The reaction was stopped by addition of 1M H_2_SO_4_. The corrected OD values at 450 nm were calculated thus: OD value of well with antigen minus OD value of well without antigen. The Sample to Positive (S/P) ratio was then determined for each sample.

#### Indirect immunofluorescence assay

The indirect immunofluorescence assay was performed using DUGV IbAr1792 infected- and non-infected Vero E6 cells according to Hartlaub et al [13]. The fixed cells were flooded with serum (1:50 diluted) after a blocking step and left to incubate for 1 hour at 37°C. Subsequently, fluorescein-labelled anti-bovine IgG conjugate was added and the slide incubated for 1 hour at 37°C after which fluorescence signal evaluation was done. Sera which gave distinct fluorescence without any signs in the corresponding non-infected Vero E6 cells were recorded positive.

#### Micro-virus neutralization test

This test was carried out in 96-well microplates with SW13 cell monolayers according to Hartlaub et al [13]. Initially, serially diluted test sera were incubated with 100 TCID_50_ DUGV (IbAr1792). These were subsequently transferred in duplicates to plates with SW13 cells. The plates were incubated for 7 days before being stained with crystal violet for observation of cytopathic effect (CPE). The virus neutralizing titers were determined using Behrens and Kaerber formula. A positive score was recorded for sera with no CPE at ≥ 1:10 dilution.

#### Interpretation of DUGV serology result

The results were interpreted based on the level of agreement among the three tests performed as earlier described for CCHFV. If all the tests gave the same outcomes (positive or negative), it was interpreted as such. However, unresolved discordant results (after repeated testing) were recorded as doubtful.

### Statistical analysis

Data were entered into the Statistical Package for Social Sciences software, version 22 (SPSS, Illinois, USA). Univariate analysis (Chi-square test) was performed and odds ratios (ORs) with 95% confidence interval (CI) calculated to assess the relationship between the seroprevalence of CCHFV and DUGV, respectively with each risk factor. The ORs were determined with respect to a reference category as indicated in the respective tables. Multivariable unconditional logistic regression was used to determine predictors for both CCHFV and DUGV exposure controlling for other covariates at p < 0.20. Collinearity among predictors was tested using the Chi-square test for binomial variables. Also, interactions between significant variables using forward stepwise method was done. The level of significance was determined at p ≤ 0.05.

## Results

Seroprevalence rates of 71.9% (631/877) and 52.8% (451/854) were obtained for CCHFV and DUGV, respectively among sampled cattle. Additionally, CCHFV seroprevalence was highest in January (90.0%) and lowest in August (20.0%), while DUGV seroprevalence was highest in July (96.4%) but lowest in January (20.0%) (Table 1). Across the months, three peaks were observed for both CCHFV and DUGV IgG prevalence.

**Table 1:** Univariate analysis of monthly distribution of CCHFV and DUGV IgG prevalence among cattle in Kwara State, Nigeria.

Month	CCHFV				DUGV			
	Freq.	Positive (%)	OR (95% CI)	P-value	Freq.	Positive (%)	OR (95% CI)	P-value
January	10	9 (90.0)	9.0 (1.0–80.9)	0.0562	10	2 (20.0)	0.6 (0.1–3.2)	0.6927
February	79	61 (77.2)	3.4 (1.4–8.4)	0.0149*	66	48 (72.7)	5.9 (2.3–15.4)	0.0002*
March	365	293 (80.3)	4.1 (1.9–8.9)	0.0006*	368	125 (34.0)	1.1 (0.5–2.6)	0.8402
May	58	48 (82.8)	4.8 (1.8–13.1)	0.0023*	55	29 (52.7)	2.5 (1.0–6.4)	0.0684
July	26	19 (73.1)	2.7 (0.9–8.5)	0.1005	28	27 (96.4)	60.0 (7.0–513.0)	<0.0001*
August	5	1 (20.0)	0.3 (0.0–2.5)	0.3457	5	4 (80.0)	8.9 (0.9–91.1)	0.0586
September	28	19 (67.9)	2.1 (0.7–6.3)	0.2772	26	20 (76.9)	7.4 (2.2–24.7)	0.0011*
October	122	86 (70.5)	2.4 (1.0–5.5)	0.0466*	112	73 (65.2)	4.1 (1.7–10.0)	0.0013*
November	156	81 (51.9)	1.1 (0.5–2.4)	1.0000	155	114 (73.5)	6.2 (2.6–14.7)	<0.0001*
December	28	14 (50.0)	1		29	9 (31.0)	1	
**Total**	**877**	**631 (71.9)**			**854**	**451 (52.8)**		

Key: CCHFV—Crimean-Congo haemorrhagic fever orthonairovirus DUGV—Dugbe orthonairovirus

Freq—Frequency OR—Odds ratio 95% CI—95% confidence interval *Significant at p < 0.05

Based on breed, N’dama cattle had the highest CCHFV seroprevalence (100.0%) followed by Red Bororo (89.5%) and Adamawa Gudali (84.4%) breeds with the least seroprevalence obtained among the Adamawa Gudali-White Fulani cross-breed (52.2%) (Table 2). However, DUGV seroprevalence was highest among White Fulani breed (74.7%) and lowest with the Adamawa Gudali (12.0%).

**Table 2:** Univariate analysis of the distribution of CCHFV and DUGV IgG prevalence among cattle breeds in Kwara State, Nigeria.

Breed	CCHFV				DUGV			
	Freq.	Positive (%)	OR (95% CI)	P-value	Freq.	Positive (%)	OR (95% CI)	P-value
SG	68	53 (77.9)	0.7 (0.3–1.3)	0.2064	66	46 (69.7)	16.9 (8.7–32.5)	<0.0001*
WF	402	260 (64.7)	0.3 (0.2–0.5)	<0.0001*	379	283 (74.7)	21.7 (13.8–34.0)	<0.0001*
B	58	34 (58.6)	0.3 (0.1–0.5)	<0.0001*	60	35 (58.3)	10.3 (5.4–19.6)	<0.0001*
RB	19	17 (89.5)	1.6 (0.3–7.1)	0.7471	19	7 (36.8)	4.3 (1.6–11.8)	0.0078*
N	4	4 (100.0)	NA	1.0000	4	2 (50.0)	7.3 (1.0–54.2)	0.0785
A	9	7 (77.8)	0.6 (0.1–3.2)	0.6369	10	4 (40.0)	4.9 (1.3–18.4)	0.0291*
Fx	5	4 (80.0)	0.7 (0.1–6.8)	0.5765	5	2 (40.0)	4.9 (0.8–30.6)	0.1198
AG x SG	24	19 (79.2)	0.7 (0.2–2.0)	0.5581	23	12 (52.2)	8.0 (3.2–19.8)	<0.0001*
WF x SG	21	15 (71.4)	0.4 (0.2–1.3)	0.1314	21	14 (66.7)	14.7 (55–39.4)	<0.0001*
AG x WF	23	12 (52.2)	0.2 (0.1–0.5)	0.0007*	25	17 (68.0)	15.6 (6.2–39.4)	<0.0001*
AG	244	206 (84.4)	1		242	29 (12.0)	1	
**Total**	**877**	**631 (71.9)**			**854**	**451 (52.8)**		

Key: CCHFV- Crimean-Congo haemorrhagic fever orthonairovirus DUGV—Dugbe orthonairovirus

Freq—Frequency OR—Odds ratio 95% CI—95% Confidence interval *Significant at p < 0.05

SG -Sokoto Gudali WF—White Fulani B—Bokolo RB—Red Bororo N—N’dama A—Azawak

Fx—Friesian AG—Adamawa Gudali x—cross NA—Not applicable

On seasonal basis, CCHFV seroprevalence was higher during the dry (80.0%) than wet season while the opposite was observed for DUGV (Wet: 67.3%; Dry: 39.4%) (Table 3). Also, cattle sampled at the abattoirs were observed to have higher CCHFV IgG prevalence than those in pastoralist farms, while DUGV seroprevalence was higher among cattle in pastoralist farms (74.9%) than in abattoirs.

**Table 3:** Risk factor analysis of the distribution of CCHFV and DUGV IgG prevalence in Kwara State, Nigeria.

Features	CCHFV				DUGV			
	Freq.	Positive (%)	OR (95% CI)	P-value	Freq.	Positive (%)	OR (95% CI)	P-value
**Season**								
Dry	455	364 (80.0)	2.3 (1.7–3.1)	<0.0001*	444	175 (39.4)	0.3 (0.2–0.4)	<0.0001*
Wet	422	267 (63.3)	1		410	276 (67.3)	1	
**Animal location**								
Abattoir	615	503 (81.8)	4.7 (3.4–6.5)	<0.0001*	595	257 (43.2)	0.3 (0.2–0.4)	<0.0001*
Farm	262	128 (48.9)	1		259	194 (74.9)	1	
**Sex**								
Male	131	49 (37.4)	0.2 (0.1–0.2)	<0.0001*	129	82 (63.6)	1.7 (1.1–2.5)	0.0096*
Female	746	582 (78.0)	1		725	369 (50.9)	1	
**Age (years)**								
< 1.6	131	47 (35.9)	0.1 (0.0–0.1)	<0.0001*	128	87 (68.0)	3.5 (2.3–5.4)	<0.0001*
1.6–2.5	86	44 (51.2)	0.2 (0.1–0.3)	<0.0001*	85	62 (72.9)	4.5 (2.6–7.6)	<0.0001*
2.6–3.5	82	58 (70.7)	0.4 (0.2–0.7)	0.0033*	78	51 (65.4)	3.1 (1.9–5.2)	<0.0001*
3.6–4.5	74	56 (75.7)	0.5 (0.3–1.0)	0.0558	71	51 (71.8)	4.2 (2.4–7.4)	<0.0001*
4.6–5.5	54	45 (83.3)	0.9 (0.4–1.9)	0.6838	54	25 (46.3)	1.4 (0.8–2.5)	0.2323
5.6–6.5	110	91 (82.7)	0.8 (0.5–1.5)	0.5433	106	50 (47.2)	1.5 (1.0–2.3)	0.0883
> 6.5	340	290 (85.3)	1		332	125 (37.7)	1	
**Body condition score**								
Poor	33	23 (69.7)	0.6 (0.1–3.2)	0.6983	34	23 (67.6)	1.7 (0.4–7.5)	0.6960
Fair	245	180 (73.5)	0.7 (0.1–3.3)	1.0000	234	157 (67.1)	1.6 (0.4–6.2)	0.4861
Good	589	420 (71.3)	0.6 (0.1–3.0)	0.7322	577	266 (46.1)	0.7 (0.2–2.6)	0.7394
Very good	10	8 (80.0)	1		9	5 (55.6)	1	
**Tick infestation (no of ticks)**								
0	56	40 (71.4)	0.9 (0.3–2.8)	1.0000	57	18 (31.6)	0.1 (0.0–0.3)	<0.0001*
1–50	690	496 (71.9)	1.0 (0.4–2.5)	1.0000	671	344 (51.3)	0.2 (0.0–0.6)	0.0009*
51–100	109	79 (72.5)	1.0 (0.4–2.8)	1.0000	104	70 (67.3)	0.3 (0.1–1.2)	0.1199
>100	22	16 (72.7)	1		22	19 (86.4)	1	
^**a**^ **Herd size (no of cattle)**								
1–25	35	4 (11.4)	0.1 (0.0–0.4)	0.0011*	37	21 (56.8)	0.1 (0.0–0.7)	0.0099*
26–50	43	26 (60.5)	1.3 (0.4–3.7)	0.7854	42	27 (64.3)	0.1 (0.0–0.9)	0.0238*
51–75	126	67 (53.2)	0.9 (0.4–2.4)	1.0000	123	99 (80.5)	0.3 (0.0–2.0)	0.3081
76–100	38	20 (52.6)	0.9 (0.3–2.7)	1.0000	40	31 (77.5)	0.2 (0.0–1.9)	0.2527
> 100	20	11 (55.0)	1		17	16 (94.1)	1	
^**a**^ **Grazing system**								
Nomadic	176	87 (49.4)	1.1 (0.6–1.8)	0.7942	177	131 (74.0)	0.9 (0.5–1.6)	0.7582
Semi-nomadic	86	41 (47.7)	1		82	63 (76.8)	1	
^**a**^ **Tick control interval (month)**								
< 3	24	12 (50.0)	0.8 (0.3–2.3)	0.8035	24	16 (66.7)	0.2 (0.1–0.8)	0.0246*
3–5	192	91 (47.4)	0.8 (0.4–1.4)	0.4161	186	135 (72.6)	0.3 (0.1–0.8)	0.0134*
> 5	46	25 (54.3)	1		49	43 (87.8)	1	
**TOTAL**	**877**	**631 (71.9)**			**854**	**451 (52.8)**		

Key: CCHFV—Crimean-Congo haemorrhagic fever orthonairovirus DUGV—Dugbe orthonairovirus Freq—Frequency

OR—Odds ratio 95% CI—95% Confidence interval

*Significant at p < 0.05

^a^ Samples from abattoir cattle

were not considered

Based on age, cattle above 6.5 years and below 1.6 years had the highest (85.3%) and lowest (35.9%) CCHFV seroprevalence, respectively while cattle within 1.6–2.5 years old and those above 6.5 years old had the highest (72.9%) and lowest (37.7%) DUGV seroprevalence rates, respectively (Table 3). Cattle with “very good” body condition score had the highest CCHFV IgG prevalence (80.0%) while those with “poor” body condition score had the highest DUGV seroprevalence (67.6%). Based on the burden of tick infestation, there was no statistically significant difference in CCHFV seroprevalence between those with high tick burden (72.7%) and those with no tick burden (71.4%). However, lower DUGV seroprevalence was associated with decrease in tick infestation burden.

Additionally, pastoralist farms with herd size ranging between 26–50 cattle had the highest CCHFV seroprevalence while those with more than 100 cattle had the highest DUGV seroprevalence. Based on tick control interval, CCHFV seroprevalence was highest in farms with >5 months tick control program (54.3%) while the least was found in farms with 3–5 months tick control program (47.4%) (Table 3). However, pastoralist farms on > 5-month tick control program had the highest DUGV seroprevalence (87.8%) while those on < 3-month program had the least (66.7%). CCHFV and DUGV IgG were detected in all the LGAs sampled (Table 4) with the highest CCHFV seroprevalence (82.4%) recorded in Ilorin West LGA and lowest (20.0%) in Ifelodun LGA. Contrariwise, DUGV IgG detection was highest in Asa LGA (97.7%) and lowest in Patigi LGA (33.3%).

**Table 4:** Univariate analysis of the distribution of CCHFV and DUGV IgG prevalence among cattle in various LGAs in Kwara State, Nigeria.

LGAs	CCHFV				DUGV			
	Freq.	Positive (%)	OR (95% CI)	P-value	Freq.	Positive (%)	OR (95% CI)	P-value
Ilorin West	556	458 (82.4)	18.7 (2.1–169.2)	0.0045*	542	210 (38.7)	0.2 (0.0–1.4)	0.0795
Patigi	14	4 (28.6)	1.6 (0.1–19.1)	1.0000	15	5 (33.3)	0.1 (0.0–1.4)	0.1273
Ilorin East	38	19 (50.0)	4.0 (0.4–39.2)	0.3508	39	35 (89.7)	2.2 (0.2–24.7)	0.4698
Ilorin South	39	16 (41.0)	2.8 (0.3–27.3)	0.6337	40	27 (67.5)	0.5(0.1–5.1)	1.0000
Offa	69	52 (75.4)	12.2 (1.3–117.2)	0.0210*	62	53 (85.5)	1.5 (0.1–14.7)	0.5664
Oyun	18	14(77.8)	14.0 (1.2–163.5)	0.0329*	16	12 (75.0)	0.8 (0.1–8.8)	1.0000
Asa	44	29 (65.9)	7.7 (0.8–75.5)	0.0671	43	42 (97.7)	10.5 (0.5–201.9)	0.1995
Moro	6	4 (66.7)	8.0 (0.5–128.0)	0.2424	6	4 (66.7)	0.5 (0.0–8.0)	1.0000
Baruten	42	16 (38.1)	2.5 (0.3–24.0)	0.6400	43	33 (76.7)	0.8 (0.1–8.3)	1.0000
Kaiama	46	18 (39.1)	2.6 (0.3–24.9)	0.6389	43	26 (60.5)	0.4 (0.0–3.7)	0.6372
Ifelodun	5	1 (20.0)	1		5	4 (80.0)	1	
**Total**	**877**	**631 (71.9)**			**854**	**451 (52.8)**		

Key: CCHFV—Crimean-Congo haemorrhagic fever orthonairovirus DUGV—Dugbe orthonairovirus

Freq—Frequency OR—Odds ratio 95% CI—95% Confidence interval

*Significant at p < 0.05

Multivariate analyses conducted to determine the association between each risk factor studied and CCHFV IgG seroprevalence and DUGV IgG seroprevalence, respectively showed that some of these risk factors were likely significant contributors to spread of the viruses (Tables 5 and 6)

**Table 5:** Multivariate analysis of association between Crimean-Congo haemorrhagic fever orthonairovirus IgG in cattle and risk factors in Kwara State, Nigeria.

Features	β	S.E.	OR	95% CI		p- value
				Lower	Upper	
**LGA**						
Ilorin West	1.544	0.370	4.685	2.268	9.678	<0.001*
Ilorin East	-0.200	0.733	0.819	0.195	3.442	0.785
Ilorin South	-2.345	1.187	0.096	0.009	0.983	0.048
Offa	0.983	0.499	2.672	1.004	7.108	0.049
Oyun	-0.394	0.507	0.674	0.249	1.822	0.437
Asa	1.435	0.473	4.198	1.661	10.608	0.002*
Moro	1.706	0.712	5.505	1.364	22.214	0.017*
Baruten	1.273	0.499	3.572	1.343	9.504	0.011*
Kaiama	1.500	1.036	4.480	0.588	34.110	0.148
Patigi	0.743	0.502	2.102	0.786	5.622	0.139
Ifelodun			1			
**Sex**						
Male	-0.600	0.278	0.549	0.318	0.947	0.031*
Female			1			
**Age (years)**						
< 1.6	-1.910	0.284	0.148	0.085	0.259	<0.001*
1.6–2.5	-1.554	0.303	0.211	0.117	0.383	<0.001*
2.6–3.5	-0.914	0.310	0.401	0.218	0.736	0.003*
3.6–4.5	-0.777	0.333	0.460	0.240	0.883	0.020*
4.6–5.5	-0.053	0.428	0.949	0.410	2.193	0.902
5.6–6.5	-0.278	0.314	0.757	0.409	1.401	0.375
> 6.5			1			
**Tick infestation score**						
0	-0.198	0.972	0.821	0.122	5.513	0.839
1–50	0.528	0.874	1.696	0.306	9.412	0.546
51–100	-0.089	0.860	0.915	0.170	4.936	0.918
> 100			1			

Key: SE—Standard error of mean OR—Odds ratio CI—Confidence interval

*Significant at p < 0.05

**Table 6:** Multivariate analysis of association between Dugbe orthonairovirus IgG in cattle and risk factors in Kwara State, Nigeria.

Features	β	S.E.	OR	95% C.I.		p-value
				Lower	Upper	
**Month**						
January	-0.332	1.324	0.718	.054	9.625	0.802
February	1.734	0.805	5.664	1.169	27.453	0.031*
March	1.868	0.860	6.474	1.201	34.906	0.030*
May	0.926	1.056	2.526	.319	20.022	0.380
July	2.264	0.875	9.623	1.731	53.494	0.010*
August	0.620	0.781	1.859	.402	8.594	0.427
September	1.963	0.856	7.124	1.330	38.169	0.022*
October	-15.418	1.010E4	0.000	0.000	0.000	8.999
November	2.269	1.459	9.674	.554	168.802	8.120
December			1.000			
**LGA**						
Ilorin west	0.243	0.512	1.275	0.468	3.475	0.635
Ilorin East	-0.169	1.026	0.844	0.113	6.307	0.869
Offa	2.658	0.719	14.273	3.487	58.425	<0.001*
Oyun	1.024	0.595	2.786	0.868	8.936	0.085
Asa	2.308	0.747	10.053	2.327	43.437	0.002*
Moro	1.612	0.983	5.011	0.730	34.377	0.101
Baruten	20.372	1.010E4	7.038E8	0.000	0.000	0.998
Kaiama	2.183	1.346	8.874	0.634	124.152	0.105
Patigi	0.935	0.513	2.546	0.932	6.959	0.068
Ifelodun			1.000			
**Cattle breed**						
SG	0.606	0.572	1.834	0.597	5.630	0.289
WF	0.238	0.673	1.269	0.339	4.751	0.723
B	-0.572	0.788	0.564	0.120	2.643	0.468
RB	-2.136	0.538	0.118	0.041	0.339	<0.001*
N	0.438	0.496	1.549	0.587	4.093	0.377
A	-0.320	0.577	0.726	0.234	2.251	0.579
Fx	0.133	0.868	1.143	0.208	6.264	0.878
WF x AG	-0.575	1.155	0.563	0.059	5.407	0.618
SG x AG	-0.456	0.871	0.634	0.115	3.495	0.600
WF x SG	-0.532	1.200	0.587	0.056	6.167	0.657
AG			1.000			
**Sex**						
Male	-0.651	0.305	0.522	0.287	0.948	0.033*
Female			1.000			

Key: SE—Standard error of mean OR—Odds ratio CI—Confidence interval

*Significant at p < 0.05

Furthermore, the results of this study revealed that 37.9% (314/829) of the cattle were co-exposed to CCHFV and DUGV, 34.5% (286/829) were only exposed to CCHFV, 14.8% (123/829) were only exposed to DUGV, while 12.8% (106/829) were not exposed to any of the two viruses (Table 7).

**Table 7:** Co-exposure of cattle to CCHFV and DUGV in Kwara State, Nigeria.

		DUGV		^b^Total
		Positive (%)	Negative (%)	
**CCHFV**	**Positive (%)**	314 (52.3)	286 (47.7)	**600**
	**Negative (%)**	123 (53.7)	106 (46.3)	**229**
^b^ **Total**		**437**	**392**	**829**

Key: ^b^ Only sera tested for both CCHFV and DUGV were considered

Odds ratio = 0.946 95% Confidence interval = 0.697–1.284 p-value = 0.756

## Discussion

Serological evidence of circulation of CCHFV and DUGV among domestic ruminants has been documented in several African countries including Egypt, Nigeria, Senegal, Central African Republic, South Africa, Sudan, Uganda, Niger Republic, Kenya, Mauritania and Zambia [2,14,15,16,17,18,19]. The detection of CCHFV antibodies in domestic animals has been reported to be valuable since it provides early evidence of circulating virus, identifies the location of CCHFV foci, and highlights a potential and increased risk for human infection [20]. In the present study, high seroprevalence rates of 71.9% (631/877) and 52.8% (451/854) were obtained for CCHFV and DUGV, respectively in tested cattle. This finding indicates that approximately one of every three cattle in the study area is co-exposed to CCHFV and DUGV, thus establishing endemicity of the two viruses.

Additionally, the 71.9% CCHFV seroprevalence obtained for cattle in this study is higher than the 10–50% previously reported in Sokoto, Oyo and Benue States [17] and 57.1% in Borno State [21]. It is also higher than the 67%, 66%, 57.7%, 28% and 8.4% obtained in Mauritania [22], Mali [23], Niger Republic [16], South Africa [24] and Zambia [19], respectively but lower than 74% reported in Cameroun [25] and 79.1% in Afghanistan (Engil District) [26]. The CCHFV seroprevalence variations observed in States located in different geographical regions of Nigeria is consistent with existing reports of spatial variation of CCHFV seroprevalence in livestock within a single country [27,28,29]. However, the variation could also be explained by the differences in serological assays used.

The paucity of information on sero-epidemiology of DUGV globally limits our discussion to the works of Burt et al [2] and Guilherme et al [15] who reported 5.2% and 70% DUGV seroprevalence among cattle in South Africa and Central African Republic, respectively. Whereas these two studies involved the use of mouse brain-derived DUGV antigen or its sucrose-acetone extract, our DUGV seroprevalence investigation, which is the most recent study on the virus in Africa, is more reliable as it is based on a recombinant DUGV nucleoprotein ELISA, and highly specific iIFA and mVNT.

The high CCHFV and DUGV seroprevalence obtained in the study area might be due to abundance of competent vectors [30], and high livestock trading activity, as Kwara State is a major transit location in the country through which ruminants from northern Nigeria and neighbouring countries are routed to the southwestern region. Additionally, the season of the year and duration of studies might also account for the seroprevalence differences compared with previous reports. Furthermore, we observed that CCHFV seroprevalence in cattle was higher compared with similar studies conducted in small ruminants (sheep and goats) in Nigeria [17]. We propose that cattle could be one of the major livestock maintaining vectors of CCHFV and DUGV, thus ensuring their persistence in human dwellings. Adult *Hyalomma* and *Amblyomma* ticks have been observed to have preference for large-sized animals such as cattle [30,31]. This study showed a significant difference (direct proportional relationship) between tick burden and DUGV seroprevalence but not with CCHFV seroprevalence as the tick burden on cattle did not determine seropositivity rate. To unravel this discrepancy, there is a need to study the decay pattern of CCHFV antibodies in exposed animals. Furthermore, this study observed that as the age of cattle increased, CCHFV seroprevalence also increased. Guilherme et al [15] and Zohaib et al [32] reported higher CCHFV seroprevalence in older cattle than in younger ones in Central African Republic and Pakistan, respectively. However, our study observed that the DUGV seroprevalence in cattle > 6.5 years was significantly lower than that in animals < 4.5 years old (p < 0.0001).

In this study, three seroprevalence peaks each were observed for CCHFV and DUGV during the year (CCHFV: January, May and October; DUGV: February, July and November), and this correlates with the findings of Bukbuk et al [21] who also reported three peaks of CCHFV seroprevalence in Borno State, northeast Nigeria. Generally, the variations in CCHFV and DUGV seroprevalence peaks across the months of the year as observed suggest that the means of spread (mainly tick vectors) as well as the impact of environmental determinants vary considerably. Additionally, univariate analysis showed that the IgG prevalence rates obtained in December for each of CCHFV and DUGV were significantly lower from those of CCHFV in February, March, May and October (p ≤ 0.0466), and DUGV in February, July, September, October and November (p ≤ 0.0013) (Table 1). However, there is a limitation to this interpretation as sampling was conducted once in each location. Moreover, univariate analysis of CCHFV seasonal seroprevalence indicated that cattle were 2.3 times more exposed during the dry than rainy season (p < 0.0001). Bukbuk et al [21] also reported high CCHFV seroprevalence (63.1%) during the dry season in Borno State. However, our study revealed significant differences in seasonal exposure of cattle to CCHFV and DUGV as DUGV seroprevalance was higher during the wet than the dry season while the reverse was obtained for CCHFV. This finding can be attributed to the interplay of environmental factors such as relative humidity, temperature and rainfall which determine the multiplication and abundance of the tick vectors of these viruses. It has been reported that the multiplication and survival of the principal vector of CCHFV (*Hyalomma* ticks) is enhanced during the dry season with low to moderate humidity and high temperature [33,34]. However, *Amblyomma* species, the major vector of DUGV, is often more abundant during the wet season when there is moderate humidity and low temperature [30].

Our findings further reveal that all breeds of cattle used for this study were exposed to CCHFV and DUGV. However, analysis indicated significantly higher (p ≤ 0.0007) CCHFV IgG prevalence among Adamawa Gudali breed compared to each of White Fulani, Bokolo and Adamawa Gudali/White Fulani cross breeds. This observation suggests that Adamawa Gudali cattle were more exposed to CCHFV infection. During sampling, more *Hyalomma* ticks were found on brown- to dark-colored breeds of cattle including Adamawa Gudali, Red Bororo, N’dama and their cross breeds. This might be the reason for the higher CCHFV seroprevalence observed in this breed. Contrariwise, the DUGV seroprevalence obtained among Adamawa Gudali breed was significantly lower than that observed in other cattle breeds studied (except for N’dama and Friesian cross breed) (p ≤ 0.0291). This suggests that light-colored breeds of cattle were more exposed to DUGV, as the highest DUGV seroprevalence was observed among White Fulani and Sokoto Gudali breeds of cattle. We observed that light-colored breeds of cattle were highly infested with *Amblyomma variegatum* (the principal host of DUGV) as compared with other cattle breed. This might be the reason for the high DUGV seroprevalence in these cattle breeds.

Additionally, this study revealed that there was significantly higher level of DUGV exposure among cattle sampled on the pastoralists’ farms (i.e., those reared in the study locations) than those slaughtered at the abattoirs (i.e., those either reared within or outside the study location, Kwara State). Daodu et al [30] reported that cattle from farms were more infested with DUGV-positive *Amblyomma variegatum* than those from the abattoirs. In another vein, CCHFV seroprevalence was observed to be significantly higher (p < 0.0001) among cattle in abattoirs than on farms. It is expected that the overall picture of CCHFV seroprevalence of cattle reared on pastoralists’ farms in Kwara State will be comparable to the same animals slaughtered at the abattoirs. Hence, it is possible that the variation in CCHFV seroprevalence rates obtained for cattle from abattoirs and pastoralist’ farms might have been contributed by cattle reared outside the study location but slaughtered within Kwara State. Further studies focusing on these other cattle sources are needed to investigate this possibility and highlight CCHFV impact in both humans and animals. Furthermore, univariate analysis indicated that while CCHFV seroprevalence was higher in female cattle sampled than in males, the reverse was observed for DUGV seroprevalence. The reason for this difference is unknown since cattle of both sexes were equally exposed to the same environment and vectors.

Based on tick infestation level, a significantly higher (p ≤ 0.0009) DUGV seroprevalence was obtained among cattle infested with > 100 tick count compared to those with ≤ 50 ticks. This suggests that cattle with high level of tick infestation (> 100 ticks) are more likely to be exposed to DUGV and is consistent with the result obtained for DUGV prevalence based on tick control program in this study (Table 3). Pastoralist farms using tick control program with more than five months interval were observed to have significantly higher (p ≤ 0.0246) DUGV seroprevalence than those utilizing a program with < 5 months interval. However, no difference in CCHFV seroprevalence was obtained when tick control intervals were considered. This suggests that CCHFV seropositivity is independent of tick control program which inadvertently reduces tick population on cattle.

Our findings further revealed that cattle herd size could play a significant role in exposure of cattle to CCHFV and DUGV as farms with > 100 animals were observed to be more exposed to these arboviruses than those with ≤ 25 cattle (Table 3). In sub-Saharan Africa, it is difficult to control ticks on farms with large herd size probably because most of them operate semi-nomadic or nomadic system of management using old tick control methods like hand-picking of ticks. The CCHFV seroprevalence obtained in Ifelodun LGA of Kwara State was significantly lower (p ≤ 0.0329) than that in Ilorin West, Offa and Oyun LGAs (Table 4) and this might be due to low activity of the CCHFV tick vector in this LGA. Interestingly, the highest DUGV seroprevalence obtained in Asa LGA in this study corroborates the earlier report of Daodu et al [27] who showed that Asa LGA is a hotspot for DUGV infections in Kwara State.

Overall, the results of multivariate analysis indicate that local government area, sex, age (years) and tick infestation score were the risk factors that exerted significant effects on the seroprevalence of CCHFV in cattle (p < 0.05). Also, DUGV seroprevalence in cattle was significantly determined by risk factors such as month of the year, location, cattle breed and sex (p < 0.05).

## Conclusion

This study reports high antibody seroprevalence against Crimean-Congo haemorrhagic fever and Dugbe orthonairoviruses in Kwara State, Nigeria. The risk factors associated with occurrence of infections with the two viruses in cattle were highlighted. To our knowledge, this study represents the first comprehensive epidemiological survey for Dugbe orthonairovirus in Africa since its first discovery in 1964 in Nigeria. As CCHFV and DUGV are classified as Biosafety Levels 3 and 4 pathogens, respectively, continuous monitoring of these high-consequence arboviruses in animals and investigation of their impact on public health, especially among occupationally at-risk humans in Nigeria, is imperative.
